# Digitalization of health care in low- and middle-income countries

**DOI:** 10.2471/BLT.24.291643

**Published:** 2024-12-03

**Authors:** Dominique J Monlezun, Lillian Omutoko, Patience Oduor, Donald Kokonya, John Rayel, Claudia Sotomayor, Maria Ines Girault, María Elizabeth De los Ríos Uriarte, Oleg Sinyavskiy, Timothy Aksamit, Sagar B Dugani, Alberto Garcia, Colleen Gallagher

**Affiliations:** a Division of Hospital Internal Medicine, Mayo Clinic, 200 First Street SW, Rochester, MN 55905, United States of America (USA).; b Department of Educational Management, Policy and Curriculum Studies, University of Nairobi, Nairobi, Kenya.; c Clinical Trials Unit, University of Global Health Equity, Kigali, Rwanda.; d School of Medicine, Masinde Muliro University, Kakamega, Kenya.; e College of Science, Bicol University, Legazpi City, Philippines.; f Pellegrino Center for Clinical Bioethics, Georgetown University, Washington DC, USA.; g Faculty of Bioethics, Universidad Anahuac México, Mexico City, Mexico.; h Department of Public Health, Asfendiyarov Kazakh National Medical University, Almaty, Kazakhstan.; i Division of Pulmonary Medicine and Critical Care Medicine, Mayo Clinic, Rochester, USA.; j School of Bioethics, Ateneo Pontificio Regina Apostolorum, Rome, Italy.

## Abstract

The rising incidence of noncommunicable diseases, combined with the costs of mitigating climate change, sovereign debt and regional conflicts, is undermining global health security and threatening progress towards achieving the sustainable development goals of the United Nations. The negative impact of these polycrises is disproportionately borne by low- and middle-income countries, which have the highest disease burden and lowest health-care spending. Health digitalization is emerging as a promising countermeasure, accelerated by artificial intelligence (AI) software and quantum computing hardware. We provide a multisector critical analysis of the three key enablers – governance, infrastructure and security – of the responsible AI-enabled digitalization for safe, affordable, equitable and sustainable health-care systems in low- and middle-income countries. We consider leading use cases in public–private partnerships, democratized sovereign AI and embedded human security. Our analysis proposes that these use cases demonstrate how digital AI-accelerated global health may be advanced as human-centred managed strategic competition. We conducted our analysis through an inclusive range of theoretical perspectives and practical experience spanning academia, industry and practice across the world. We provide recommendations for the responsible management of the key enablers to accelerate global health for all. We anticipate that this paper will be useful for public health decision-makers, both in low- and middle-income countries leading local health digitalization, and in high-income countries supporting this transaction through their technologies, funding and knowledge exchange.

## Résumé

L’incidence croissante des maladies non transmissibles, associée aux coûts de l’atténuation du changement climatique, à la dette souveraine et aux conflits régionaux, compromet la sécurité sanitaire mondiale et menace l’avancement de la réalisation des objectifs de développement durable des Nations unies. L’impact négatif de ces crises multiples touche de manière disproportionnée les pays à revenu faible et intermédiaire, qui présentent la charge de morbidité la plus élevée et les dépenses de santé les plus faibles. La numérisation de la santé apparaît comme une contre-mesure prometteuse, accélérée par les logiciels d’intelligence artificielle (IA) et le matériel d’informatique quantique. Nous fournissons une analyse critique multisectorielle des trois principaux catalyseurs (gouvernance, infrastructure et sécurité) de la numérisation responsable basée sur l’IA pour des systèmes de soins de santé sûrs, abordables, équitables et durables dans les pays à revenu faible et intermédiaire. Nous examinons les principaux cas d’utilisation dans les partenariats public-privé, l’IA souveraine démocratisée et la sécurité humaine intégrée. Notre analyse suggère que ces cas d’utilisation démontrent la capacité des services numériques mondiaux de santé accélérés par l’IA à progresser en tant que concurrence stratégique gérée centrée sur l’humain. Nous avons mené notre analyse à partir d’un large éventail de perspectives théoriques et d’expériences pratiques couvrant le monde universitaire, le secteur médical et les cabinets médicaux à travers le monde. Nous formulons des recommandations pour une gestion responsable des facteurs clés permettant d’améliorer la santé mondiale pour tous. Nous sommes d’avis que ce document sera utile aux décideurs en matière de santé publique, à la fois dans les pays à revenu faible et intermédiaire qui promeuvent la numérisation de la santé à l’échelle locale, ainsi que dans les pays à revenu élevé qui soutiennent cette transaction par leurs technologies, un financement et l’échange de connaissances.

## Resumen

La creciente incidencia de las enfermedades no transmisibles, junto con los costes de la mitigación del cambio climático, la deuda soberana y los conflictos regionales, amenaza la seguridad sanitaria mundial y los avances hacia la consecución de los Objetivos de Desarrollo Sostenible de las Naciones Unidas. Los países de ingresos bajos y medios, que tienen la mayor carga de morbilidad y el menor gasto sanitario, asumen de forma desproporcionada el impacto negativo de estas policrisis. La digitalización de la sanidad se perfila como una prometedora contramedida, acelerada por el software de inteligencia artificial (IA) y el hardware de computación cuántica. Se ofrece un análisis crítico multisectorial de los tres factores clave: gobernanza, infraestructura y seguridad, de la digitalización responsable impulsada por la IA para lograr sistemas de atención sanitaria seguros, asequibles, equitativos y sostenibles en los países de ingresos bajos y medios. Se estudian los principales casos de uso en las asociaciones público-privadas, la IA soberana democratizada y la seguridad humana integrada. El análisis propone que estos casos de uso demuestren cómo la salud mundial acelerada por la IA digital puede avanzar como competencia estratégica, gestionada y centrada en el ser humano. El análisis se realizó desde varias perspectivas teóricas y experiencias prácticas que abarcan el mundo académico, la industria y la práctica en todo el mundo. Se presentan recomendaciones para la gestión responsable de los principales factores que aceleran la salud mundial para todos. Se prevé que este documento sea de utilidad para los responsables de tomar decisiones en materia de salud pública, tanto en los países de ingresos bajos y medios que lideran la digitalización sanitaria local, como en los países de ingresos altos que apoyan este proceso a través de sus tecnologías, financiación e intercambio de conocimientos.

## ملخص

إن ارتفاع معدلات انتشار الأمراض غير المعدية، فضلاً عن تكاليف التخفيف من التغير المناخي، والديون السيادية، والصراعات الإقليمية، يضعف الأمن الصحي العالمي ويهدد التقدم نحو تحقيق أهداف التنمية المستدامة للأمم المتحدة. إن الدول ذات الدخل المنخفض والمتوسط، والتي تعاني من أعلى عبء للأمراض وأقل إنفاق على الرعاية الصحية، تتحمل التأثير السلبي لهذه الأزمات المتعددة بشكل غير متناسب. وتبرز الرقمنة الصحية كإجراء مضاد مبشر، والتي تسارعت وتيرة انتشارها بفضل برامج الذكاء الاصطناعي (AI) وأجهزة الحوسبة الكمية. نحن نقدم تحليلاً نقديًا متعدد القطاعات للعوامل الثلاثة الرئيسية (الحوكمة، والبنية الأساسية، والأمن) المساعدة للرقمنة المسؤولة والمدعومة بالذكاء الاصطناعي من أجل أنظمة رعاية صحية آمنة، وبأسعار معقولة، وعادلة، ومستدامة في الدول ذات الدخل المنخفض والمتوسط. وندرس حالات الاستخدام الرائدة في الشراكات بين القطاعين العام والخاص، والذكاء الاصطناعي السيادي الديمقراطي، والأمن البشري المُضمّن. يقترح تحليلنا أن حالات الاستخدام هذه توضح كيف يمكن الارتقاء بالصحة العالمية المتسارعة بالذكاء الاصطناعي الرقمي كمنافسة استراتيجية مُدارة تركز على الإنسان. لقد أجرينا تحليلنا من خلال مجموعة شاملة من وجهات النظر النظرية، والخبرة العملية، التي تمتد عبر الأوساط الأكاديمية، والصناعة، والممارسة في جميع أنحاء العالم. ونحن نقدم توصيات للإدارة المسؤولة للعوامل المساعدة الرئيسية التي تسمح بتسريع الصحة العالمية للجميع. ونتوقع أن تكون هذه الورقة مفيدة لصناع القرار في مجال الصحة العامة، في كل من الدول ذات الدخل المنخفض والدخل المتوسطة التي تقود الرقمنة الصحية المحلية، وفي الدول ذات الدخل المرتفع التي تدعم هذه المهمة من خلال التقنيات، والتمويل، والتبادل المعرفي لديها.

## 摘要

随着非传染性疾病发病率的上升，以及为缓解气候变化、主权债务和区域冲突而耗费的成本增加，全球卫生安全遭到破坏，实现联合国可持续发展目标的进展也有所受阻。在面对这些重重危机时，疾病负担最重但医疗保健支出却最低的中低收入国家受到了不同程度的不利影响。在人工智能 (AI) 软件和量子计算硬件的推动下，卫生数字化转型开始出现，并被视为一种极具前景的应对措施。我们对中低收入国家由负责任 AI 驱动的数字化转型的三个关键推动因素——治理、基础设施和安全——进行了多部门批判性分析，以构建安全、公平且负担得起的可持续医疗保健系统。我们考虑了在公私合作、主权 AI 民主化和嵌入式人类安全方面的主要用例。我们的分析表明，这些用例展示了可如何推动实施以数字 AI 促进全球健康的措施，并秉持以人为本的原则来开展有管控的战略竞争。基于源自世界各地学术界、工业界和惯例的广泛理论观点和实践经验，我们展开了分析。我们就如何负责任地管理关键推动因素提出了建议，以期加快全球实现全民健康的步伐。我们期望本文能为公共卫生决策者提供一些有用信息，无论是在引领当地实现卫生数字化转型的中低收入国家，还是在通过技术、资金和知识交流来支持此类转型的高收入国家。

## Резюме

Рост заболеваемости неинфекционными болезнями в сочетании с расходами на смягчение последствий изменения климата, суверенным долгом и региональными конфликтами подрывает глобальную безопасность в области здравоохранения и ставит под угрозу прогресс в достижении целей в области устойчивого развития, поставленных Организацией Объединенных Наций. Негативное воздействие этих поликризисов в непропорционально большой степени ложится на страны с низким и средним уровнем дохода, которые несут наибольшее бремя заболеваний и меньше всего тратят на здравоохранение. Цифровизация в сфере здравоохранения становится перспективной мерой противодействия, ускоряемой программным обеспечением искусственного интеллекта (ИИ) и аппаратными средствами квантовых вычислений. В статье представлен многоотраслевой критический анализ трех ключевых факторов (управления, инфраструктуры и безопасности) для внедрения надежной цифровизации с использованием ИИ в целях создания безопасных, доступных, справедливых и устойчивых систем здравоохранения в странах с низким и средним уровнем дохода. Рассмотрены ведущие примеры использования в государственно-частном партнерстве, демократизированном суверенном ИИ и внедренной безопасности личности. По результатам анализа эти примеры использования демонстрируют, как цифровые технологии ИИ, ускоряющие развитие глобального здравоохранения, могут использоваться в качестве управляемой стратегической конкуренции, ориентированной на человека. При проведении анализа использовался широкий спектр теоретических взглядов и практического опыта, охватывающий научные, промышленные и практические круги по всему миру. Представлены рекомендации по ответственному управлению ключевыми факторами, способствующими ускорению глобального здравоохранения для всех. Ожидаем, что данный документ будет полезен для лиц, принимающих решения в области общественного здравоохранения, как в странах с низким и средним уровнем дохода, которые возглавляют процесс цифровизации здравоохранения на местах, так и в странах с высоким уровнем дохода, которые поддерживают эту операцию с помощью своих технологий, финансирования и обмена знаниями.

## Introduction

The rising incidence of noncommunicable diseases, combined with the costs of mitigating the effects of climate change, sovereign debt, human capital migration and regional conflicts, is undermining global health security and threatening progress towards achieving the sustainable development goals (SDGs).^1^^,^^2^ The effects of these polycrises are disproportionately borne by low- and middle-income countries, which have the lowest health-care spending, the highest proportions of noncommunicable disease mortality and workforce shortage, and the fastest growth in smartphone use.^3^^,^^4^ A promising and growing response to these issues is health digitalization, which is the integration of smart devices, computers, internet and other digital technologies with health ecosystems. 

Because many low- and middle-income countries currently use only a small fraction (around 5%) of their health data,^3^ health digitalization in low- and middle-income countries in the African Region alone could produce health cost savings of up to 11 billion United States dollars (US$) (around 15%) by 2030 through more personalized, effective, efficient and equitable care.^4^ Health digitalization may allow the reinvestment of billions of cost savings to boost health-care access and outcomes through optimized telehealth (remote patient–provider encounters and monitoring); self-care (digital disease prevention, diagnostics and support); paperless data (electronic health records, staff communication and digital assistants); and decision-intelligent systems (for clinicians, administrators and payors). As more people connect to the internet of things (that is, the interconnection of digital devices embedded in everyday objects) and the larger global digital ecosystem (the digital communication network of individuals, institutions, companies and governments), the increased number of users means more data and smarter insights from artificial intelligence (AI). National health systems, including local health-care systems, public health agencies, and partnering institutions, companies and governments, are part of this data explosion as health-care records, communications, financing and administration are increasingly digitalized.

In 2021, the World Health Organization (WHO) recognized that AI-augmented digital health was a proven accelerator for improving health outcomes through the SDGs, translating such data into actionable insights for more efficient health and policy management at the individual, public and global health levels.^5^^,^^6^ The health sector globally is progressively benefitting from the technological evolution of AI digitalization, accelerated by the intensifying international competition between leading companies and governments.^1^ Generative AI alone is expected to add more than US$ 10 trillion to the global economy, sufficient to cover the entire cost of global health.^7^^,^^8^ However, nine of the world’s top 10 technology companies are located in the United States of America (USA), with China and the USA accounting for over two thirds of AI investments worldwide.^1^ Similarly, China and the USA dominate funding and advances in quantum computing – the use of quantum physics to solve more complex problems faster than traditional digital computers – necessary for the exponential evolution of AI.^9^ However, as this competition for the critical strategic assets of AI and quantum computing determines the global balance of great power, it threatens to divide the global digital ecosystem and weaken collaboration in global health digitalization.^1^^,^^10^^–^^12^

These trends in AI shape the global horizontal competition between political economic blocs of countries as well as the vertical competition between their companies and institutions (the meta-determinants of health). As the United Nations (UN) Secretary-General warned, “divides are deepening” between democratic and so-called autocratic countries, as well as between countries of higher- and lower-income classifications.^13^ The UN Secretary-General therefore called for a return to the global consensus of the SDGs, anchored in “the dignity and worth of every person” and oriented ultimately to “the common good.” Without managing this strategic competition through such consensus, health digitalization in low- and middle-income countries may be delayed or even endangered as conflict replaces cooperation. 

Our international team assessed three key enablers – governance, infrastructure and security – for responsible AI-facilitated digitalization in the health sectors of low- and middle-income countries to optimize safety, sustainability, affordability and equity. Failure to harness and manage these enablers carries significant risks, including (i) undermining societal trust and engagement; (ii) worsening the digital divide; and (iii) eroding health security. To mitigate such risks, we attempt to bring an inclusive range of theoretical perspectives and practical experiences spanning the academic, industrial and health sectors across the world. We anticipate that this paper will be useful for decision-makers leading public health digitalization in low- and middle-income countries (and to their partners in high-income countries, supporting these aims through technologies, funding and knowledge exchange). Our ultimate purpose is to provide an integral vision of, and pragmatic recommendations for, managing these enablers to accelerate global health.

## Governance

The 2021 *Ethics and governance of artificial intelligence for health: WHO guidance* defines health governance as the collective mechanisms used by governments and institutions for strategic guidance and operational rules to achieve national health policy objectives.^14^ Digital health governance is a key enabler for responsible digitalization because the national health systems of low- and middle-income countries must align their partners under a shared strategic objective, realized through a common operational plan and backed by an enforceable set of fair rules for how partners are to work together to achieve this. Effective governance is particularly useful for syncing efforts from the diverse partners required to comprehensively address health determinants to maximize global health benefit, namely: individual (behaviours and biology, understood through multi-omics); environmental (water, air and housing), commercial (company research, products, services, marketing and pricing); digital (literacy, accessibility and personalization); and political and economic determinants.^1^^,^^15^^,^^16^ The political and economic determinants are also considered as the meta-determinants, as power and resource allocation societally can amplify the positive or negative impact of the other health determinants. 

There exists a proliferation of proposed governance frameworks and conveners of these frameworks, mostly from democracies or global institutions focused on human rights, including the UN, the UN Educational, Scientific and Cultural Organization, the Institute of Electrical and Electronics Engineers, the Group of Seven, the European Union, the Organisation of Economic Development and Co-operation, and the World Economic Forum.^17^^–^^22^ However, the majority of practical daily governance in AI digitalization comes from American companies with over US$ 33 billion (over two thirds) controlling global stakes in digital AI chips and software, and US$ 411 million in quantum computing (around one third, double that of the next closest competitor), operating according to their own rights-based governance frameworks (with substantive overlap with the above multilateral frameworks).^23^^–^^25^ Generally, the abovementioned frameworks support public–private partnerships as a concrete model of successful governance, matching global digital capabilities with local health needs.

The WHO governance model supports such a collaborative approach to responsible health-care digitalization.^5^ WHO calls for a global person-centric digital health ecosystem spanning these partnership networks as an essential enabling factor to realize the ultimate vision of health for all. As a pioneering early adopter and partner, India launched the Global Initiative on Digital Health in 2023.^26^ WHO coordinates and aligns multisector stakeholders according to needed resources, best practices and global standards; meanwhile, India adopts these practices and resources for health digitalization according to its local needs.^6^ WHO facilitates the digital health knowledge exchange, transformation toolbox (digital tools), country resource portal (mapping digital health resources) and country needs tracker according to the related second and third strategic objectives in its 2020–2025 digital health strategy.^5^ Although WHO launched the virtual platform for the Initiative, a promising template for other low- and middle-income countries, funding for and integration of the platform in the national digital health system of India is lagging. This partnership builds on the country’s rapid health digitalization (accelerated by the coronavirus disease 2019 (COVID-19) pandemic) to expand its national digital health ecosystem, including through the Ayushman Bharat Digital Mission for a country-wide electronic health record with digital health identification number for every citizen.^27^

Parallel private efforts include India’s Lupin Digital Health that launched its digital health platform, Lyfe, as the country’s first digital cardiac rehabilitation programme spanning remote monitoring, clinical care and public health prevention.^28^ Through its collaboration with the American College of Cardiology, assisting the knowledge exchange of best practices and physician certification, the programme has already demonstrated improved treatment adherence and reductions in blood pressure, sugar and cholesterol (such that India’s Central Drugs Standard Control Organization granted it a Class D Medical Device License).^29^

Deepening collaborative networks are also facilitating health digitalization with the integration of responsible precision medicine and precision public health, that is, uniting multi-omics, geospatial and predictive analytics with big data (large amounts of highly varied data from many different sources that may be processed rapidly) and AI real-time analyses, enabling patients and populations to affordably and reliably obtain the right intervention at the right time.^30^ In January 2024, the Bill & Melinda Gates Foundation partnered with the WHO Regional Office for Africa to accelerate funding for precision public health efforts to predict, prevent, track and mitigate disease spread, with an initial emphasis on malaria eradication.^31^ Such efforts may also help to efficiently match limited national health resources with local health needs to maximize net public benefit through expanded digitalization.

## Infrastructure

Digital infrastructure is the technological enabler of health digitalization in low- and middle-income countries, as governance is its organizational enabler. This infrastructure refers to the network of physical and software assets that collectively enable data flow (including data centres, storage, cloud and security), required to move digital products and services from providers to users.^1^ Examples include the USA-based Mayo Clinic Platform, which extended its international federated health data network to the WHO Eastern Mediterranean Region and Brazil by 2024, keeping partners’ data safe locally while sharing the AI-accelerated insights gleaned from them globally.^32^ With a similar model, TriNetX created the world’s largest end-to-end health data architecture and platform.^33^ The infrastructure overseen by this private American company coordinates over 220 health-care organizations in 30 countries, including low- and middle-income countries in Africa, South America and Asia, expanding clinical trial opportunities along with precision public health insights. Ghana and Zimbabwe were early collaborators and shared de-identified electronic health records in return for access to resources. There are significant risks to having a private company provide governance and infrastructure (e.g. changing ownership, solvency, public accountability and national regulations), but the largest and most used digital health platforms globally are still led and provisioned by such companies.^1^ Users generally appear to perceive them as generating greater value, affordability, functionality and reliability compared with institutions and governments, largely because vertical competition speeds the creative destruction of lower performing and less trusted platform companies.

There exist many other partnerships in the expansion of the digital infrastructure of low- and middle-income countries: USA-based Microsoft and the Egyptian government are improving food and health security by digital app-based boosting of agriculture productivity;^34^ and the African start-up, Access Afya, is expanding telehealth services to remote and lower-income patients, enabled by the mobile banking service M-Pesa. Digital infrastructures are also becoming greener, as seen with Microsoft and G42 (the leading AI company of the United Arab Emirates),^35^ who partnered with the Kenyan government in 2024 to build a geothermal-powered data centre in Olkaria at a cost of US$ 1 billion. The data centre allowed the expansion of more sustainable, reliable and affordable computing, essential for processing and analysing the growing digitalized health systems of low- and middle-income countries. Such cost savings can then be used to provide more people with universal health coverage, allowing them to access those digital AI-augmented health services. The USA-based Tufts University published their free online African Leapfrog Index to facilitate this infrastructure roll-out using the lessons learnt from such initial case studies spanning six countries of the continent.^36^ Along with governance, the index highlights infrastructure as a key digitalization enabler. The index also shows how to map the specific components of digitalization and its progress at the country level (including improved energy reliability, government protection of individual digital freedoms, digital provider competition and digital money exchange).

Digital infrastructure is finally becoming smarter. American AI and digital companies led by Nvidia are increasingly democratizing decolonized sovereign AI through the cloud, that is, provisioning the digital technologies that enable countries to transparently and affordably build, own, operate and locate on-site their home-grown quantum AI digital infrastructure.^37^ As more countries seek to avoid becoming overly reliant on China or the USA in their digital AI competition, India is again emerging as a key leader in such digitalization through its sovereign AI push backed by Nvidia. China provides a different infrastructure model in its Belt and Road Initiative.^38^^,^^39^ The government-driven initiative reportedly promotes national sovereignty by expanded infrastructure options for countries. By 2023, the initiative may have created history’s largest international physical and digital infrastructure, with cooperative agreements spanning 150 countries. Chinese researchers report that the programme “boosted the expenditure of public health and effectively spurred economic growth.” Some low- and middle-income countries appear to be safeguarding their investments by partnering with local and international digital infrastructure providers from both China and the USA, benefitting from the competition of both countries for their partnerships.^39^

## Human security

Human security is the final enabler of health digitalization in low- and middle-income countries, the strategic organizing principle facilitating responsible governance and infrastructure to attain and maintain this. The UN’s 2012 General Assembly Resolution 66/290 defined human security as “freedom from fear … and want.”^40^ Taking a holistic approach to health determinants, human security entails “people-centred, comprehensive, context-specific and prevention-oriented responses that strengthen the protection and empowerment of all people and all communities.”^40^ The UN recognizes the proven value of applying human security, which “can significantly enhance actions … to fully realize the transformative promise” of the SDGs.^41^ Human security also entails its constitutive elements of effective health data security (protections for people’s digital information from theft, corruption and unauthorized access) and health security (the proactive and reactive measures mitigating the negative effects of health threats). Health security partnerships in health digitalization are progressively transforming African public health, especially through optimized prediction (disease outbreaks from different data streams), health-care accessibility (better diagnostics and telemedicine) and workforce shortages (through democratized AI).^42^

The textbook *Responsible artificial intelligence re-engineering the global public health ecosystem* seeks to advance this approach for low- and middle-income countries.^1^ Emblematic use cases in the textbook include integrated methods and education. The AI-driven computational ethics and policy analysis applies propensity score translational statistics augmented by quantum Bayesian machine learning for causal health inference, cost–effectiveness and personalist social contract ethics.^1^^,^^43^^,^^44^ The approach features propensity score analysis that is boosted with Bayesian and machine learning to identify higher risk patient populations and their health risk factors. These results then feed into a cost–effectiveness analysis of proposed interventions for these groups, which in turn informs a human-centred approach to pluralistic global bioethics to enumerate concrete recommendations for their care. The approach is engineered to be end-to-end, adaptive, automated and embedded in the digital infrastructures of health ecosystems to aid in responsible digitalization and health AI operations (in contrast to competing methods, which typically focus in silos on clinical, cost or policy aspects). The approach has since been used to inform real-time operational, policy and strategic recommendations, especially in equitable nutrition, cardiovascular disease, cancer prevention and care tailored for lower income communities. In terms of education or workforce capacity-building in human security, the Mexico-based Anahuac University Masters in Global Bioethics has educated health professionals and policy-makers on four continents (primarily from low- and middle-income countries in the African and South-East Asia regions) on this comprehensive approach for local health solutions.

## AI-enabled global health 

We have described how the three key enablers of governance, infrastructure and security may provide substantive and pragmatic guidance for navigating responsible health digitalization in low- and middle-income countries amid human-centred managed strategic competition.^1^ This approach maps global health as an interconnected three-level ecosystem to facilitate the productive cooperation of diverse partners. These levels include political economic interoperability of diverse regimes (democracies and autocracies); data interoperability (data architectures); and moral interoperability (through core common values about human dignity, rights and the common good across belief systems and their embodied cultures). This comprehensive approach seeks to develop the model of the World Bank for responsible health digitalization in low- and middle-income countries: moving from capacity-building through health data digitization, to piloting fragmented digital health applications, to scaling mature digital health solutions that are person-centred, embedded and integrated throughout a health ecosystem.^3^

The major next steps in advancing such digitalization may be emblematically seen in human-centred quantum health AI, requiring and enabling stronger partnerships and financing between countries of higher and lower incomes.^45^ A leading use case is the Open Quantum Institute of the European Council for Nuclear Research as a multilateral science diplomacy initiative, internationally empowering more open and inclusive quantum computing to advance the SDGs, especially in low- and middle-income countries.^46^ This public–private partnership seeks to identify, prove and scale quantum AI use cases, including enhancing the sustainability of global food systems (quantum optimization), and early diagnosis and imaging accuracy (quantum machine learning). To further democratize quantum AI-accelerated digitalization, Nvidia is expanding quantum AI access through its hybrid quantum-classical platform.^47^ Reliance Jio, India’s largest telecommunication company, is working to expand energy-efficient AI digital infrastructure to its 450 million customers, for which Nvidia provides the AI supercomputing service.^48^ Simultaneously, IBM partnered with Cleveland Clinic and Cleveland’s state government in 2023 to launch the first on-site, hybrid, cloud-enabled quantum health AI computer accelerating drug discovery and population health tools, which can then be diffused through partnerships between countries of higher and lower incomes.^49^ IBM’s collaboration with Kenya and South Africa leverages such resources not only to build local educational capacity and solutions (now extending to 15 universities across nine African countries for research and workforce education)^50^ but to advance the science that informs and scales up such health-care innovations at the local level. The areas included are public health countermeasures in climate change, clean water, sustainable food supplies and financial inclusion. For example, extending cloud-based services to low- and middle-income countries for lower costs compared with building this infrastructure themselves.

These trends and use cases, along with our experiences, inform the following recommendations for responsible digitalization in low-and middle-income countries: (i) foster competitive and mutually beneficial local and international public–private partnerships, for example, encouraging local health systems to consider different vendors with varied digitalization offerings and finance models, and to select and modify such partnerships as required; (ii) enhance human-centred digitalization co-design and co-deployment, with local communities guiding how digital technologies are created, applied, governed and revised so they are safe, affordable, useful and integrated with local workflows; (iii) prioritize agile, scalable and end-to-end technology innovations, empowering rapid local iteration in concert with digital providers, including in federated and swarm architectures, seamless integration, offline capacities, security and governance; (iv) accelerate continuous local capacity-building in data architecture, management, workforce training, innovation, financing and automation; and (v) safeguard individual rights, cultural autonomy and the unifying strategic objective of health for all.

From lessons learnt and promising next steps, we have shown how such use cases and recommendations in health digitalization in low- and middle-income countries may inform useful theoretical templates and practical advances towards more sustainable, efficient and equitable health for each person. We envisage how this aim can be scaled worldwide to achieve health for all, raised to the exponential power of quantum AI-accelerated digitalization, and anchored in humanity’s common good that bounds the strategic competition shaping these technologies.
